# The Association of Non-obscene Socially Inappropriate Behavior With Attention-Deficit/Hyperactivity Disorder Symptoms, Conduct Problems, and Risky Decision Making in a Large Sample of Adolescents

**DOI:** 10.3389/fpsyt.2019.00660

**Published:** 2019-09-13

**Authors:** Valerie Brandt, Julia Kerner auch Koerner, Emma Palmer-Cooper

**Affiliations:** ^1^Department of Psychology, Centre for Innovation in Mental Health, University of Southampton, Southampton, United Kingdom; ^2^Educational Psychology, Helmut-Schmidt-University, Hamburg, Germany; ^3^Center for Individual Development and Adaptive Education of Children at Risk (IDeA), Frankfurt am Main, Germany

**Keywords:** Tourette syndrome, attention deficit and hyperactivity disorder, conduct (behavioral) problems, non-obscene socially inappropriate behavior, Cambridge Gambling Task

## Abstract

Non-obscene socially inappropriate behavior (NOSI) is recognized as part of the tic disorder spectrum but has received little attention from researchers to date. A study in 87 patients with Tourette syndrome showed that comorbid attention-deficit/hyperactivity disorder (ADHD) and conduct disorder were also associated with an increase in socially inappropriate behavior. This study used data from the Millennium Cohort Study to investigate the relationship between NOSI and emotional symptoms, conduct problems, and hyperactivity/inattention as assessed by the Strengths and Difficulties Questionnaire (SDQ) in 1,280 youths, aged 14 years. Furthermore, the relationship between NOSI and decision-making processes as assessed by the Cambridge Gambling Task (CGT) was investigated. Hyperactivity/inattention and conduct problems were significantly associated with NOSI; emotional problems were not. Risk taking was significantly associated with misbehaving in lessons but not with being rude or noisy in public. The results replicate and confirm the association of NOSI with ADHD and conduct problems in a large sample, although it should be stressed that the size of the association was small. The results also suggest that some inappropriate behaviors are related to risk-taking behavior, while others are not.

## Introduction

Non-obscene socially inappropriate behavior (NOSI), such as making inappropriate comments about a person’s appearance (e.g., weight, height) or performing inappropriate actions (e.g., pulling the fire alarm) (1), currently has no generally agreed-upon operational definitio. Very little research has been conducted into this phenomenon, and this has been limited to the field of tic disorders (TDs) (1, 2).

TDs are childhood-onset neuropsychiatric disorders (3); tics are repetitive but not stereotyped movements (e.g., eye blinking, jumping) or vocalizations (e.g., coughing, barking) that occur out of context. However, beyond the diagnostic criteria, TDs are multifaceted disorders that can encompass a range of different phenomena, such as echopraxia and echolalia, i.e., repeating other people’s movements or sounds (4, 5); premonitory urges, i.e., uncomfortable sensory phenomena accompanying tics (6–10); and copropraxia and coprolalia, i.e., involuntary obscene gestures or obscene sounds, words, or sentences (11). While coprolalia is very salient and frequently portrayed by the media as characteristic for TDs, the prevalence in Tourette syndrome (TS) is currently estimated at 10–33% (11–15) and depends on the sample (16).

But not all inappropriate behavior can be classified as “coprophilia” (coprolalia or copropraxia) (1, 17). Kurlan et al. (1) investigated NOSI in 87 patients with TS. A significant number of patients reported insulting others as a habit (22%), more typically family members than strangers, 5% reported making socially inappropriate comments, and 14% reported having performed socially inappropriate actions (1). The incidence of NOSI in TDs in studies with sample sizes < 100 was estimated at approximately 25–50% (1, 2). It has been speculated that NOSI might be related to increased or decreased sensitivity to social cues (18–20) and poor decision making (21). It was also found that NOSI is significantly related with attentional problems or attention-deficit/hyperactivity disorder (ADHD) and conduct problems (1, 2) as well as with obsessions (2). Moreover, socially disinhibited behavior is highly heritable in patients with TS (22).

ADHD is characterized by age-inappropriate and impairing levels of inattention, impulsivity, and hyperactivity (3). Socially inappropriate behavior is not a core symptom of ADHD (3); however, it often co-occurs with ADHD symptoms. Children and adolescents with ADHD tend to demonstrate inappropriate social behaviors, such as intrusive, commanding, and hostile behavior with peers (23). High rates of aggressive behavior and rule breaking, relative to typically developing peers, have been reported (24). Moreover, socially inappropriate behaviors appear to be related to low impulse control in children with ADHD (25). This suggests that NOSI may be part of a more general impairment of impulse control or decision making, although it is unclear whether NOSI may have different underlying mechanisms in different disorders.

Conduct disorder (CD) is characterized by socially inappropriate behavior, such as aggressive behavior, damage to property, and rule breaking (3, 26). Children with conduct problems tend to make riskier decisions than control participants (27, 28) and are more likely to be impulsive and display sensation seeking and antisocial behavior as youths (29). Having both ADHD and conduct problems appears to further exacerbate risky decision making in children (30) [for a review on decision making in ADHD and CD, see Ref. (31)].

The current study investigates the relationship between indicators of NOSI, e.g., complaints about being rude or noisy in public, and symptoms of hyperactivity/inattention as well as conduct problems, as assessed by the Strengths and Difficulties Questionnaire (SDQ). Furthermore, the relationship between NOSI and risky decision making, as assessed by the Cambridge Gambling Task (CGT), is explored.

## Methods

### Participants

The data from the 2015 Millennium Cohort Study (MCS) sweep were used (32). SDQ data were available for 11,323 participants (5,696 males), aged 14 years. Information about complaints for being rude or noisy in public was available for *n* = 11,192 youths. Out of the sample, 1,467 youths (13.1%) had been complained about for being rude or noisy in public, and the number of complaints was given for *n* = 1,280 participants (683 males). Information about misbehaving in class was available for *n* = 11,192 youths. Available data for individual analyses can vary slightly due to missing data; therefore, each N is reported in **Tables 1** and **2**.

**Table 1:** Association between NOSI indicators and indicators of hyperactivity, conduct, and emotional problems.

Rude/noisy in public	SDQ hyperactivity/inattention	SDQ conduct problems	SDQ emotional problems	Sex
N	1,244	1,244	1,244	1,280
*rho (p)*	.17 (< .001)	.17 (< .001)	.07 (.02)	−.007 (.82)
95% CI	.11 to.22	.11 to.22	.01 to.13	−.06 to.05
Partial *rho* controlled for sex	.17 (< .001)	.17 (< .001)	.07 (.016)	
**Misbehavior in lessons**				
N	11,021	11,029	11,026	11,358
*rho (p)*	.28 (*p* < .001)	.24 (*p* < .001)	.01 (*p* = .35)	−.16 (< .001)
95% CI	.27 to.31	.23 to.27	−.01 to.03	−.18 to −.14
Partial *rho* controlled for sex	.26 (*p* < .001)	.24 (*p* < .001)	−.03 (*p* < .001)	
95% CI	.25 to.29	.23 to.27	−.05 to −.01	

The table displays correlation coefficients for the association between indicators of non-obscene socially inappropriate behavior (NOSI) and SDQ subscales hyperactivity/inattention, conduct problems, and emotional problems. Partial correlations are controlled for sex. CI, confidence interval.

**Table 2:** Association between NOSI indicators and CGT subscales.

Rude/noisy in public	CGT risk taking	CGT quality of decision making	CGT overall proportional bet
N	1,217	1,217	1,217
*rho (p)*	.05 (.10)	−.05 (.096)	.05 (.063)
95% CI	−.01 to.11	−.11 to.01	−.01 to.11
**Misbehavior in lessons**			
N	10,653	10,654	10,654
*rho (p)*	.14 (< .001)	−.09 (< .001)	.13 (< .001)
95% CI	.12 to.16	−.11 to −.07	.11 to.15
Partial *rho* controlled for sex, SDQ hyperactivity/inattention, and SDQ conduct disorder	.10 (.001)	−.03 (.274)	.10 (.001)
95% CI	.08 to.12	−.05 to −.01	.08 to.12
**Sex**			
N	10,718	10,719	10,719
*rho (p)*	−.25 (< .001)	.02 (.019)	−.22 (< .001)
95% CI	−.27 to −.23	.001 to.04	−.23 to −.20

The table displays correlation coefficients for the association between indicators of NOSI and three subscales of the Cambridge Gambling Task (CGT).

### Measures

**Socially inappropriate behaviors.** NOSI was assessed as the self-reported frequency with which the cohort member had been complained about for being rude or noisy in public (number of complaints) and misbehaving in lessons (1–4; 1 = all the time and 4 = never; for this study, the scale was recoded so that higher numbers represent higher incidents of misbehavior).

**SDQ hyperactivity/inattention problems.** The parent-rated subscale inattention/hyperactivity of the SDQ (33), an internationally used and validated screening questionnaire to assess mental and behavioral strengths and difficulties in 3- to 16-year olds, was used at all measurement occasions to assess ADHD symptoms. The SDQ is widely used for measuring ADHD symptoms (34) and shows high correlations with other scales assessing ADHD symptoms, for instance, the Conners Scale (35) or the Child Behavior Checklist (CBCL) (36). The SDQ is better able to distinguish between children with and without ADHD than the CBCL with 118 items (11 for attention problems) (37). The five-item inattention/hyperactivity subscale sums up ratings of ADHD-related behavior and has good internal consistency (average Cronbach’s α = .87, maximum = 10 points). The items for the subscale are “restless, overactive, cannot stay still for long,” “constantly fidgeting or squirming,” “easily distracted, concentration wanders,” “thinks things out before acting,” and “sees tasks through to the end, good attention span.”

**SDQ conduct problems.** The parent-rated five-item conduct problem subscale sums up ratings of conduct-related behavior and has lower internal consistency than other subscales (average Cronbach’s α = 0.67, maximum = 10 points). The items for this subscale are “often has temper tantrums or hot tempers,” “generally obedient,” “often fights with other children,” “often lies or cheats,” and “steals from home, school or elsewhere.”

Every item of the SDQ is rated on a three-point Likert scale: “not true” (0), “somewhat true” (1), and “certainly true” (2). Positively worded items are reverse-scored. The possible range is 0–10 (*M* = 3.2 in the norm sample) (38). Teachers carried out the assessment. According to Woerner and colleagues (39), the critical cutoff for clinical significance is greater than or equal to seven raw score points (39).

**SDQ emotional problems.** The subscale “emotional problems” of the SDQ was used as a control variable in the current study to ensure that the associations found with NOSI and the CGT were specific to conduct problems and hyperactivity/inattention. The subscale also consists of five items, assessing emotional problems, such as the tendency to worry or be fearful.

**Figure 1:**
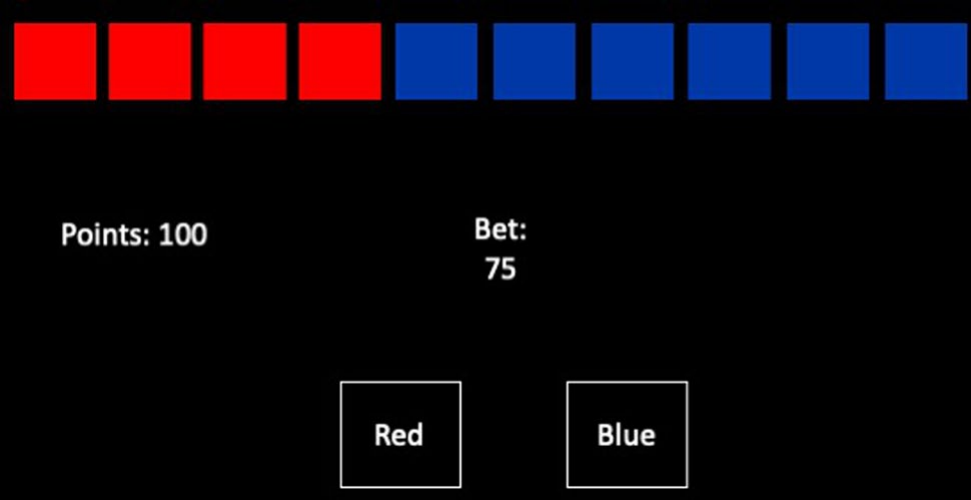
The Cambridge Gambling Task. Participants have to place a bet on whether a token is hidden in the red or the blue boxes at the top. Box color is chosen using the selection boxes at the bottom of the screen. If the bet is correct, the number of points bet is added to their score.

**Cambridge Gambling Task.** The CGT (40) is a computerized assessment of risk taking and decision making and behavior, where no learning is involved (see **Figure 1**).

In each trial, there are 10 boxes presented at the top of the screen, where the ratio of red to blue boxes varies across trials. Participants are told that there is a token hidden in one of the boxes, and the task involves selecting which color box the token is hidden in. Box color is chosen using the selection boxes at the bottom of the screen. The task takes up to 18 min to complete.

Participants start the task with 100 points and in each trial must “bet” points on their decision about which box will contain the token. If they are correct, the number of points bet is added to their score. If they are wrong, the number of points bet is removed from their score. Participants choose the number of points to bet by pressing the “Bet” circle in the center of the screen when it shows the value they wish to bet; this value incrementally changes. Once a selection and a bet are made, the token is revealed, and points are altered according to the selection and the bet made.

In line with the original task design, points won by the end of the task were not associated with any financial value (40). Evidence demonstrates that financial rewards are not the only factor to influence risk taking, where impulsive processing can guide decision making with the expectation of any objective or subjective reward (41). The CGT employs a point reward system and provides performance feedback, both of which have been linked to reward processes in the brain (42). Individuals diagnosed with disordered gambling make riskier decisions than healthy controls when completing the CGT (43), demonstrating that the task is sensitive to differences in risky decision making in the absence of financial reward. Research has also demonstrated that pathological gamblers show behavioral shifts towards risk taking (44), as do individuals with attentional disorders (45).

Scores calculated from this task and reported in the MCS data include: risk taking, quality of decision making, decision time, risk adjustment, delay aversion, and the overall proportional bet. For the purpose of this study, we were interested in risk taking, the quality of decision making, and the proportional size of the bet participants placed. Quality of decision making is calculated using participants’ judgments about which color box the token is hidden in, where a higher proportion of trials in which the more likely outcome is chosen indicates better decision making. Risk taking is calculated using the mean proportion of current points that the participant bets on each trial, when the more likely outcome is selected.

### Statistical Analysis

The incidence of having been complained about for being rude or noisy in public or not (yes/no) and its relationship with the SDQ subscales was assessed with a rare events logistic regression in R 3.6.1 (46), due to its low base rate in the sample (13%). Rare events logistic regression takes the whole sample into account. Analysis code in **Supplementary Materials**.

Normal distributions were tested using Shapiro–Wilk tests. Neither frequency of being rude or noisy in public (0.18, *p* < .001) nor frequency of misbehaving in class (.79, *p* < .001) was normally distributed. Furthermore, the SDQ conduct problems (.85, *p* < .001), SDQ hyperactivity/inattention (.94, *p* < .001), and emotional symptoms (.85, *p* < .001) were also non-normally distributed. While CGT risk taking (1.0, *p* = .19) and overall proportional bet (1.0, *p* = .19) were normally distributed, CGT quality of decision making was not (.84, *p* < .001). CGT risk-taking values ranged from.05 to.95, indicating a wide range of risk-taking decisions. Therefore, Spearman’s rank correlation coefficient *rho* was used to assess the association between the indicators of NOSI and the three SDQ subscales as well as the CGT subscales. Partial, non-parametric correlations were used to control for the effects of sex. Correlation coefficients were Fisher-transformed to calculate confidence intervals. All tests of significance were two-tailed. Analyses were run in SPSS 24 (47).

## Results

Both hyperactivity/inattention (β = .08, SE = .01, z = 6.02, *p* < .001) and conduct problems (β = .16, SE = .02, z = 8.13, *p* < .001; intercept β = −2.25, SE = .05, z = −44.65, *p* < .001) were significantly associated with having been complained about for being rude or noisy in public, while emotional problems were negatively associated with having been complained about (β = −.08, SE = .02, z = −5.37, *p* < .001).

Mean number of complaints was 5.5 (*SD* = 19.16, range = 1–300). Mean misbehavior in class was 1.6 (*SD* = .64). Associations between frequency of complaints about being rude or noisy in public, as well as misbehavior in lessons, and the SDQ are reported in **Table 1**.

Risk taking, quality of decision making, and the size of the overall proportional bet as assessed by the CGT were significantly associated with misbehaving in lessons but not with frequency of being rude or noisy in public (**Table 2**). Exploratory analyses showed that the difference between the correlations was significant for CGT risk taking (z = −2.67, *p* = .008) and overall proportional bet (z = −2.37, *p* = .018) but not for quality of decision making (z = 1.18, *p* = .24). With Bonferroni correction for three *post hoc* *t*-tests, the only difference that remained significant was for the CGT risk-taking subscale.

## Discussion

The results of this study showed that there was a significant relationship of both hyperactivity/inattention and conduct problems with indicators of NOSI. The results are in line with previous findings (1, 2), confirming an association of attentional and conduct problems with NOSI, in a large non-clinical sample of adolescents. The results are presumably independent of TDs, although data on the latter were not available from this cohort. However, the results also show that the relationship is small. The results indicate that NOSI could be considered a cross-disorder phenomenon and is likely not a form of complex motor or vocal tic (1). This is also in line with findings from a heritability study, showing that socially disinhibited tics were associated with comorbid Obsessive Compulsive Disorder (OSD) and ADHD (22). Even though coprophenomena have been shown to be associated with NOSI in TDs (1, 2), they might be different phenomena. It is more likely that there is a common underlying mechanism that facilitates both coprophenomena and NOSI that could be related to impulse control, decision-making processes, sensitivity to social cues, (18, 20, 21) or compulsive tendencies (1, 2). It has been suggested that difficulties in distinguishing between the mental states of self and others may be associated with socially inappropriate behaviors in TDs (18, 20, 48). However, experimental data will be needed to confirm this association directly and whether the same mechanism is applicable to ADHD and conduct problems. Which mechanisms are found to play into NOSI may also depend on how NOSI is operationalized. In this study, the frequency of being rude or noisy in public and the frequency of misbehaving in lessons were taken as indicators of NOSI.

Interestingly, the association between attentional problems and conduct problems was larger with misbehavior in class than with being rude or noisy in public. Two explanations are possible. First, it is possible that misbehavior in class is a milder indicator for NOSI than rudeness in public. Rudeness in public may tap into a different construct that may be closer to insulting others or even to coprophenomena. Second, it is possible that misbehaving in class is taken as an indicator for hyperactivity and conduct problems when they are assessed. School records are often used to aid diagnosis of ADHD, for instance. Thus, a more formal definition of NOSI is necessary to avoid circularity between assessing inattention/conduct problems and assessing NOSI as an independent construct. In this study, circularity is unlikely because parent-reported SDQ scores were used, rather than diagnoses. Classroom behavior is unlikely to play an important role in the parents’ assessment of their child’s ADHD and conduct problem symptoms because the questionnaire does not assess them. The NOSI indicators were self-reported by the adolescents and therefore independent from the SDQ scores. Furthermore, the experimental data on decision making are independent of both of them. Interestingly, the data show that parent-rated problems and risky decisions in the CGT only predicted risky behavior in public and school to a small extent.

Decision-making processes were explored in this study as a possible underlying mechanism for NOSI. Risk taking in the CGT was significantly associated with misbehaving in class, even when attentional and conduct problems were controlled for. In contrast, lower quality of decision making was mainly explained by higher attentional and conduct problems. Interestingly, being rude or noisy in public was not related to risky decision-making processes. Again, the results highlight the necessity for a more formal operationalization of NOSI. The tendency to make risky decisions could impact NOSI actions, such as damaging objects, pulling a fire alarm, or acting out in class, while NOSI verbalizations, such as insulting others or being rude, may be influenced by other underlying processes, such as sensitivity to social cues (21). Alternatively, there could be a difference in the processes affecting NOSI in a familiar setting and NOSI in a public setting. It has been shown that NOSI occurs more commonly in familiar settings than in public settings in TDs (1). To facilitate future research, it would be useful to formally operationalize NOSI, independent of disorders, and perhaps to define a clinically relevant cutoff that would allow research in clinical and subclinical populations.

In conclusion, indicators of NOSI were found to be significantly associated with both attention and conduct problems in adolescents, suggesting a cross-diagnostic phenomenon. Risky decision-making processes were weakly associated with NOSI in a familiar setting but not in a public setting.

A strength of this study is the large sample size and that the indicators of hyperactivity/inattention and conduct problems were continuous rather than categorical (yes/no). The main limitation of this study is that only certain indicators of NOSI could be used, i.e., being rude or noisy in public and misbehaving in lessons. Overall, in order to research NOSI more widely, it would be helpful to define NOSI more clearly and to define at what point it becomes clinically relevant. For instance, misbehaving in lessons could be considered a normal, that is, subclinical, expression of NOSI as long as it does not lead to serious consequences, such as expulsion from school.

## Data Availability

The datasets generated for this study will not be made publicly available. The data are openly available from the Millennium Cohort Study, UCL.

## Ethics Statement

Ethical review and approval was not required for the study on human participants in accordance with the local legislation and institutional requirements. Written informed consent from the participants’ legal guardian/next of kin was not required to participate in this study in accordance with the national legislation and the institutional requirements.

## Author Contributions

VB and EP-C conceptualized the study. VB analyzed the data. VB and JK drafted the manuscript. EP-C drafted the CGT methods section and critically revised the manuscript. Each author contributed different aspects of expertise: VB on TDs and NOSI, JK on ADHD, and EP-C on the CGT and CD.

## Conflict of Interest Statement

The authors declare that the research was conducted in the absence of any commercial or financial relationships that could be construed as a potential conflict of interest.
