# Supplementary material for: The Association of Non-obscene Socially Inappropriate Behavior With Attention-Deficit/Hyperactivity Disorder Symptoms, Conduct Problems, and Risky Decision Making in a Large Sample of Adolescents
Source: Front Psychiatry. 2019 Sep 13;10:660. doi: 10.3389/fpsyt.2019.00660 (PMC6753840; doi:10.3389/fpsyt.2019.00660)
Supplement: Supplementary file 1 [file DataSheet_1.docx]

**Supplementary materials**

Analysis script

SPSS:

******select only participants with SDQ scores**.

USE ALL.

COMPUTE filter_$=(FEBDTOT ~= -1).

VARIABLE LABELS filter_$ 'FEBDTOT ~= 0 (FILTER)'.

VALUE LABELS filter_$ 0 'Not Selected' 1 'Selected'.

FORMATS filter_$ (f1.0).

FILTER BY filter_$.

EXECUTE.

**frequency sex**.

FREQUENCIES VARIABLES=FCCSEX00

/ORDER=ANALYSIS.

**Frequency and descriptives rude in public, no filter**.

FREQUENCIES VARIABLES=FCRUDE00

/ORDER=ANALYSIS.

DESCRIPTIVES VARIABLES=FCRUDN00

/STATISTICS=MEAN STDDEV MIN MAX.

**Recoding misbehaving in lessons variable**.

RECODE FCMISB00 (1=4) (2=3) (3=2) (4=1) INTO MisbehavLessons.

VARIABLE LABELS  MisbehavLessons 'MisbehavLessonsRecode'.

EXECUTE.

**Table 1**

*nonparametric correlations**.

CORRELATIONS

  /VARIABLES=FCRUDN00 MisbehavLessons FHYPER FCONDUCT FEMOTION FCCSEX00

  /PRINT=TWOTAIL NOSIG

  /MISSING=PAIRWISE.

NONPAR CORR

  /VARIABLES=FCRUDN00 MisbehavLessons FHYPER FCONDUCT FEMOTION FCCSEX00

  /PRINT=SPEARMAN TWOTAIL NOSIG

  /MISSING=PAIRWISE.

*nonparametric partial correlations**.

NONPAR CORR FCRUDN00 FEMOTION FCONDUCT FHYPER FCCSEX00

/MISSING = LISTWISE

/MATRIX OUT(*).

RECODE rowtype_ ('RHO'='CORR') .

PARTIAL CORR FCRUDN00 FEMOTION FCONDUCT FHYPER BY FCCSEX00

/significance = twotail

/MISSING = LISTWISE

/MATRIX IN(*).

NONPAR CORR MisbehavLessons FEMOTION FCONDUCT FHYPER FCCSEX00

/MISSING = LISTWISE

/MATRIX OUT(*).

RECODE rowtype_ ('RHO'='CORR') .

PARTIAL CORR MisbehavLessons FEMOTION FCONDUCT FHYPER BY FCCSEX00

/significance = twotail

/MISSING = LISTWISE

/MATRIX IN(*).

**Table 2**

*nonparametric correlations**.

CORRELATIONS

  /VARIABLES=FCRUDN00 MisbehavLessons FCCSEX00 FCGTRISKT FCGTQOFDM FCGTOPBET

  /PRINT=TWOTAIL NOSIG

  /MISSING=PAIRWISE.

NONPAR CORR

  /VARIABLES=FCRUDN00 MisbehavLessons FCCSEX00 FCGTRISKT FCGTQOFDM FCGTOPBET

  /PRINT=SPEARMAN TWOTAIL NOSIG

  /MISSING=PAIRWISE.

*nonparametric partial correlations**.

NONPAR CORR FCRUDN00 MisbehavLessons FCGTOPBET FCGTRISKT FCGTQOFDM FCONDUCT FHYPER FCCSEX00

/MISSING = LISTWISE

/MATRIX OUT(*).

RECODE rowtype_ ('RHO'='CORR') .

PARTIAL CORR FCRUDN00 MisbehavLessons FCGTOPBET FCGTRISKT FCGTQOFDM BY FCONDUCT FHYPER FCCSEX00

/significance = twotail

/MISSING = LISTWISE

/MATRIX IN(*).

*Frequency Risk taking decisions CGT*.

FREQUENCIES VARIABLES=FCGTRISKT

/FORMAT=NOTABLE

/HISTOGRAM NORMAL

/ORDER=ANALYSIS.

R:

mydata <- mydata[FCRUDE00 >= 0]

mydata <- mydata[FCONDUCT>= 0]

mydata <- mydata[FHYPER>= 0]

mydata <- mydata[FEMOTION>= 0]

summary (z.out)

z.out <- zelig(FCRUDE00 ~ FCONDUCT + FHYPER +FEMOTION, model = "relogit", tau = NULL,

               bias.correct = TRUE,

               data = mydata)

x.out <- setx(z.out)

s.out <- sim(z.out, x = x.out)

summary(s.out)

plot(s.out)
